# Prevention of Pressure Sore Recurrence with Fat Graft: Outcome Analysis with Recurrence, Thickness, and Scar—A Pilot Study

**DOI:** 10.1055/a-2702-1486

**Published:** 2026-01-30

**Authors:** Ondřej Troup, Barbora Blažková, Milena Troupová, Adam Skalický, Inka Třešková

**Affiliations:** 1 Department of Plastic Surgery, University Hospital in Pilsen, Pilsen, Plzeň Region, Czech Republic; 2 Medical Faculty in Pilsen, Charles University, Pilsen, Prague, Czech Republic; 3 Department of Radiology, Hospital České Budějovice, České Budějovice, South Bohemian Region, Czech Republic

**Keywords:** pressure ulcer, spinal cord injuries, fat grafting, secondary prevention

## Abstract

**Background:**

Pressure ulcers are a common and debilitating complication in patients with spinal cord injuries (SCIs), often requiring reconstructive surgery. However, scarred areas remain vulnerable to recurrence. This study evaluates lipografting as a secondary prevention strategy to enhance soft tissue padding over bony prominences and reduce ulcer recurrence. Additionally, it investigates whether fat resorption rates are higher in compromised tissue.

**Methods:**

Five wheelchair-bound male patients with SCIs who had previously undergone reconstructive surgery for ischial pressure sores were included. Lipografting was performed, and soft tissue thickness was measured using ultrasonography preoperatively, immediately postoperatively, and at 3 and 12 months. Scar pliability, patient satisfaction, and fat resorption rates in the compromised area were also assessed.

**Results:**

Significant soft tissue augmentation was observed immediately postoperatively, with an average retention rate of 60.7% at 1 year. Scar pliability improved in all patients, and no new pressure ulcers developed during the follow-up period. Patients reported high satisfaction, with one noting increased tolerance for prolonged sitting.

**Conclusion:**

Lipografting appears to be a promising, minimally invasive approach for secondary prevention of pressure ulcer recurrence in SCI patients. While these findings are encouraging, further studies with larger cohorts and longer follow-up are necessary to confirm the long-term benefits of this technique.

## Introduction

Pressure ulcers are a common and debilitating complication in individuals with spinal cord injury (SCI), with a lifetime incidence of up to 95%. 1 These ulcers primarily result from prolonged immobility and impaired sensation, leading to localized shear and compression over bony prominences such as the ischial tuberosities, sacrum, and trochanters. 2 Sustained pressure impairs capillary blood flow, causing ischemia, accumulation of toxic metabolites, and eventual tissue necrosis. 3

The National Pressure Ulcer Advisory Panel (NPUAP) classifies pressure ulcers into four stages, based on the extent of tissue damage—from superficial erythema (Stage I) to full-thickness tissue loss involving muscle, bone, or joint capsule (Stage IV). 4 While early-stage ulcers can often be managed conservatively through repositioning, dressings, antimicrobials, and nutritional support, advanced stages typically require surgical intervention, including debridement and reconstruction using flaps or grafts. 5 Despite such measures, recurrence remains high, with reported rates exceeding 50% due to poor vascularity and tissue atrophy in pressure-prone regions. 6

Management of pressure ulcers is both complex and costly. In contrast, preventive strategies—although initially expensive—are shown to reduce long-term health care costs. 7 Recommendations include the use of pressure-relieving devices, moisture control, and nutritional optimization. 8 Nevertheless, the risk persists due to chronic pressure, sensory deficits, and progressive soft tissue atrophy.

We propose that lipografting could enhance tissue resilience in pressure-prone areas by increasing soft tissue volume and improving regenerative capacity. In addition to mechanical padding, adipose tissue contains stromal vascular fraction, including adipose-derived stem cells (ADSCs), which secrete angiogenic factors such as VEGF and promote neovascularization. 9 However, graft survival is contingent on adequate vascularity at the recipient site; a 1:1 ratio between fat droplets and capillaries is essential for graft integration. 10 Resorption rates after lipografting range from 25 to 75%, 11 and outcomes may be further compromised in scarred or ischemic regions.

In a 2016 study by Previnaire et al, fat grafting in previously reconstructed sites showed favorable results in most patients, though substantial resorption was observed in a minority. 12 Their reliance on caliper-based measurements limits the accuracy of these findings. In our study, we aim to improve evaluation precision using ultrasonography to assess graft retention and outcomes more reliably.

## Methods

This clinical trial was approved by the Ethical Committee of University Hospital Pilsen and the Faculty of Medicine in Pilsen, Charles University, Edvarda Benese 1128/13, 30599, Pilsen, Czech Republic, under the reference number 129/23.

The study included five patients who underwent surgery between October 2022 and April 2023. We conducted lipografting on pressure areas previously treated with reconstructive surgery to study the rate of fat resorption in these compromised regions, assess the impact on skin quality, and evaluate its potential to prevent further development of pressure ulcers. Strict inclusion criteria were applied for this study. We selected male patients aged 33 to 59 years with SCIs who were wheelchair-bound, non-smokers, and led active lifestyles. All patients had undergone at least one prior reconstructive operation for pressure ulcers, with an average of three surgeries. To maintain uniformity, we included only patients who had previously been treated for ischial pressure sores. Most of the patients had been treated for left-sided ischial pressure sores, and the time since their last reconstructive operation ranged from 4 months to 14 years ( Table 1 ). Preoperatively, ultrasonography was used to measure the soft tissue thickness in the pressure areas previously treated for deep pressure sores, and only patients with a thin soft tissue layer (less than 1 cm in thickness) were included.

**Table 1:** This table presents the demographic and clinical details of the study participants, including age, level, and age of spinal cord injury, number of previous operations for pressure sores, type of pressure sore, type of surgical procedure, and the interval from the last operation

Patient	Age	Level of injury	Age of injury	Number of operations for pressure sore	Type of pressure sore	Type of operation	Interval from last operation
1	50	Th5	24	6	Left ischiadic, left trochanteric	Primary closure, musculocutaneous flap (m. gluteus maximus, Hamstring)	7 months
2	59	Th5	25	2	Left ischiadic	Musculocutaneous flap (m. gluteus maximus, M. biceps femoris)	4 months
3	43	C7	24	1	Right ischiadic	Musculocutaneous flap (m. gluteus maximus)	13 years
4	33	Th6	19	3	Left ischiadic, sacral	Musculocutaneous flap (m. gluteus maximus)	4 years
5	54	Th10	41	3	Left ischiadic	Musculocutaneous flap (m. gluteus maximus)	4 months

Patients were hospitalized for the procedure. Fat was harvested from one side of the lower abdomen. Subcutaneous tissue was infiltrated with a tumescent solution containing 0.9% saline with adrenaline (1 mg/L). Based on previous studies highlighting the negative effect of local anesthetics on graft survival, 13 no local anesthetics were used, as the recipient area lacked sensitivity. Fat was manually aspirated using a blunt 3-hole cannula (3 mm diameter) attached to a 50-mL syringe. The amount of harvested fat ranged from 80 to 180 mL, with an average of 115 mL, depending on the requirements of the recipient site.

**Fig. 1:**
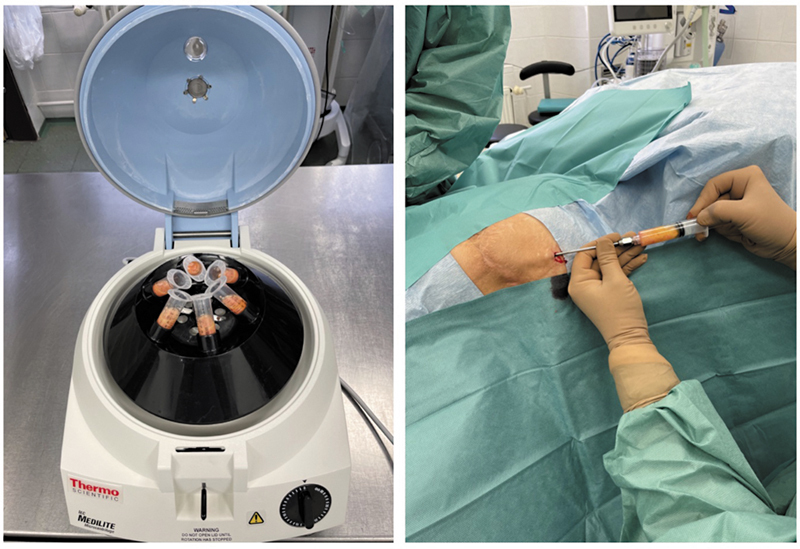
This figure illustrates the sterile processing of lipoaspirate, including its division into 10-mL syringes, centrifugation at 3,000 rpm for 3 minutes, and subsequent fat grafting using blunt cannulas for layered injection.

The lipoaspirate was processed in a sterile environment, divided into 10-mL syringes, and centrifuged at 3,000 rpm for 3 minutes. After centrifugation, the fat was cleaned of any oily components and excess fluids. Meanwhile, the recipient area was prepared, draped, and one to two small puncture incisions were made near the treatment site. Scar tissue adhesions in the recipient area were released using an 18-G needle (rigotomy). Fat grafting was performed using blunt 1-hole cannulas with a 2-mm diameter, injecting small amounts of fat in a three-dimensional pattern between layers of host tissue ( Fig. 1 ). This technique promotes graft survival and stability. 14 The amount of fat injected into each area depended on the existing soft tissue thickness; care was taken to avoid overfilling, which could result in fat necrosis due to insufficient contact with vascularized tissue. 15 The volume of grafted fat ranged from 50 to 140 mL, with an average of 81 mL. Patients were discharged the following day and advised to avoid excessive pressure on the treated area. Follow-up appointments were scheduled at 14 days postsurgery to assess healing and remove sutures. Afterward, patients were allowed to resume their usual activities.

Ultrasonography of the recipient area was performed immediately after surgery to document the increase in soft tissue thickness. Further ultrasonographic evaluations of the recipient site were conducted at 3 and 12 months postoperatively to assess the thickness of the soft tissue. The resorption rate was calculated as a percentage by comparing the postoperative thickness to the thickness at subsequent follow-up intervals. The ultrasonography probe was consistently placed in the same position without applying any pressure to ensure the reliability and consistency of the results.

To evaluate the quality of scars resulting from previous reconstructive surgery in the recipient area, we utilized the Vancouver Scar Scale (VSS), which is widely recognized as a reliable tool for scar assessment. 16 The scars were evaluated preoperatively and 12 months following lipografting. With the written consent of the patient, the photo documentation was performed in regular intervals during the follow-up period.

All procedures were performed by a single surgeon, and all ultrasonography examinations were conducted under the supervision of a specialized radiologist. The total follow-up period was 12 months.

Given the small sample size and the expected direction of change following treatment, only descriptive statistics such as mean and standard deviation were used to present the results.

## Results

No postoperative healing complications were observed during the study, either immediately after surgery or over the long-term follow-up period. All surgical wounds healed primarily, without any signs of delayed healing, infection, or dehiscence. The only minor postoperative issue noted was the presence of small, self-limiting hematomas in the liposuction donor areas. These hematomas resolved naturally within a few weeks without requiring medical intervention.

One intraoperative event was documented: A patient experienced a transient hypertensive episode during the procedure. This was promptly managed with a single dose of Tensiomin (12.5 mg), after which the patient's blood pressure stabilized, and no further complications arose. The patient remained normotensive throughout the postoperative period and was discharged in stable condition.

**Fig. 2:**
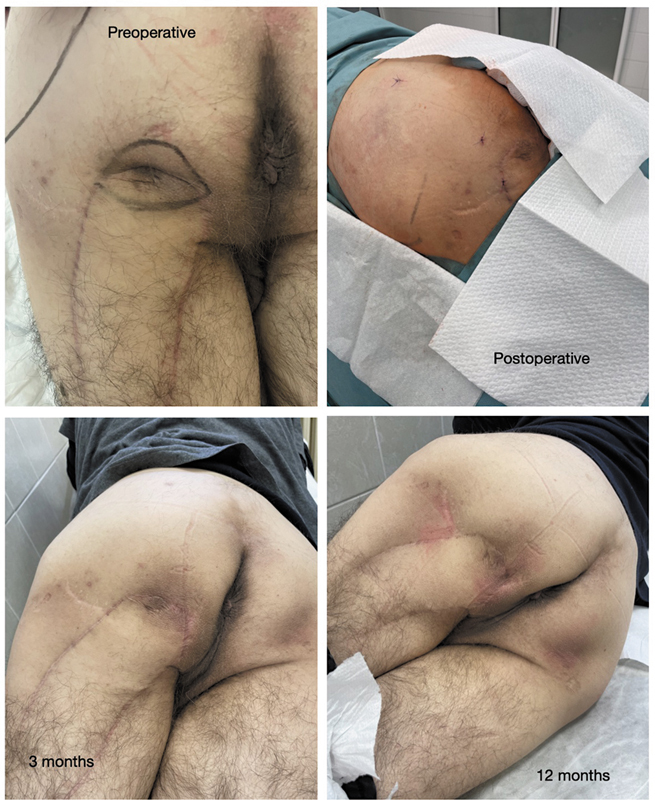
The figure presents preoperative, postoperative, 3- and 12-month images of the grafted area, demonstrating tissue augmentation and improved scar quality over time.

Throughout the entire 12-month follow-up period, no new pressure ulcers developed in the recipient areas of any patient. The skin in these regions remained intact, and patients continued their usual daily activities without interruption. Clinical observations confirmed that the grafted areas demonstrated improved tissue quality and durability against pressure-related stress ( Fig. 2 ).

### Ultrasonographic Measurements

**Fig. 3:**
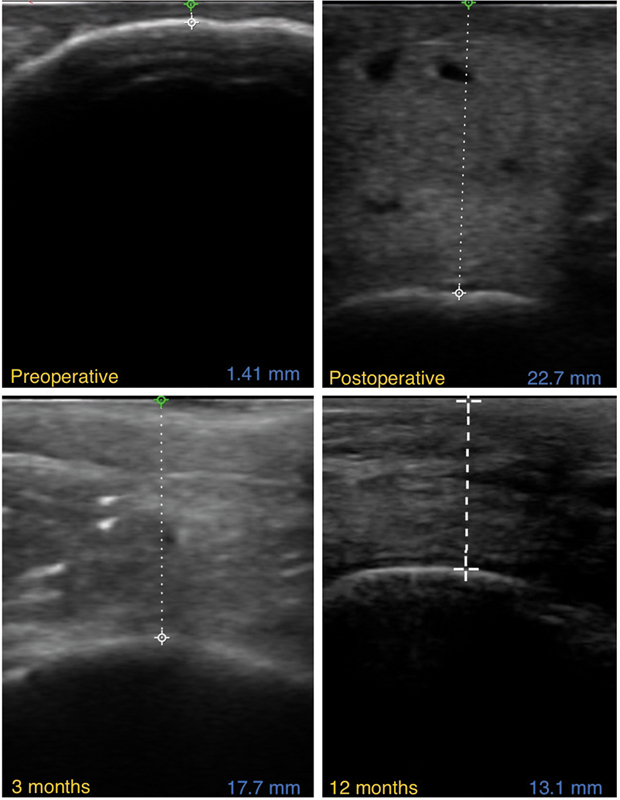
The figure shows preoperative, postoperative, 3- and 12-month ultrasonography images, illustrating initial tissue augmentation and the gradual decrease in thickness due to fat graft resorption.

Ultrasonography was performed at specific intervals to monitor changes in soft tissue thickness over the treated areas. Measurements were taken preoperatively, immediately postoperatively, and at 3 and 12 months following the procedure. The postoperative results were compared with the preoperative baseline to evaluate the degree of augmentation and the resorption of grafted fat over time ( Fig. 3 ).

### Soft Tissue Thickness over Time

**Fig. 4:**
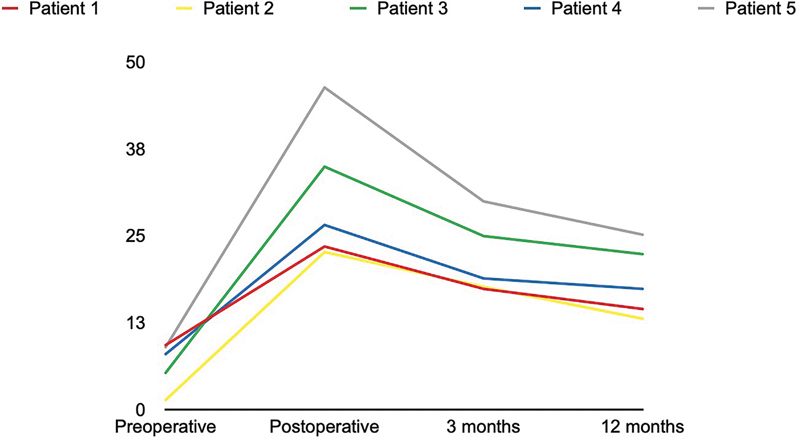
This graph shows the changes in soft tissue thickness (mm) over a timeline, with data points at preoperative, postoperative, 3 months, and 12 months for each patient. The x-axis represents the timeline, while the y-axis represents the soft tissue thickness in millimeters. A greater decrease in thickness is observed up to 3 months, with a smaller reduction after 3 months, as evidenced by the flattening of the line at the 12-month mark.

- Preoperative: The average preoperative soft tissue thickness was 6.6 ± 3.3 mm, with measurements ranging from 1.4 to 9.3 mm.
- Immediately postoperative: Postoperative thickness increased significantly, with an average of 30.8 ± 1 mm, and measurements ranged from 22.7 to 46.4 mm.
- Three-month follow-up: At 3 months, resorption was observed, and the average thickness was 21.8 ± 5.5 mm, with values ranging from 17.4 to 30 mm.
- Twelve-month follow-up: After 12 months, further resorption occurred, and the average thickness was 18.52 ± 5.2 mm, with measurements ranging from 13.1 to 25.2 mm ( Table 2 and Fig. 4 ).

**Table 2:** This table presents the soft tissue thickness measurements for each patient at four time points: Preoperative, immediately postoperative, 3 months, and 12 months

Patient	Location	Thickness of soft tissue preoperatively (mm)	Volume of lipoaspirate (mL)	Volume of graft (mL)	Thickness after operation (mm)	Thickness after 3 months (mm)	Resorption rate after 3 months (%)	Thickness after 12 months (mm)	Resorption rate after 12 months (%)
1	Left ischiadic	9.3	80	50	23.5	17.4	26%	14.5	38%
2	Left ischiadic	1.4	140	105	22.7	17.7	22%	13.1	42%
3	Right ischiadic	5.3	95	60	35	25	29%	22.4	36%
4	Left ischiadic	8	80	50	26.6	18.9	29%	17.4	34%
5	Left ischiadic	9	180	140	46.4	30	35%	25.2	46%
Mean		6.6	115	81	30.8	21.8	28.2%	18.5	39.2%
SD		3.3	–		9.97	5.52	–	5.16	–

It also includes the volume of lipoaspirate and graft used during surgery. Additionally, the table shows the fat resorption rates at 3 and 12 months postoperatively, indicating the percentage of graft volume lost over time.

### Fat Resorption Analysis

**Fig. 5:**
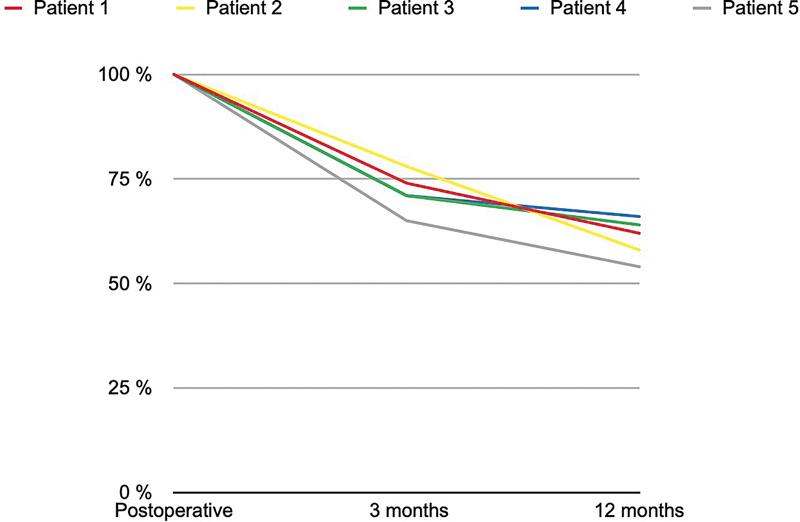
This line graph illustrates the percentage of retained fat graft volume in five individual patients over a 12-month follow-up period. The x-axis represents the timeline at three intervals: postoperative (baseline, 100%), 3 months, and 12 months. The y-axis shows the percentage of retained graft volume, starting from 100% at baseline for all patients.

- The 3-month resorption rate ranged from 22 to 35%, with a mean of 28.2 ± 4.8%, reflecting the initial phase of volume loss.
- At 12 months, the resorption rate ranged from 34 to 46%, with a mean of 39.2 ± 4.8%, indicating a continued but variable reduction in grafted fat volume.
- Despite fat loss over time, a substantial proportion of the grafted tissue remained integrated into the recipient site after 1 year, with final thickness values remaining significantly higher than preoperative measurements ( Table 2 and Fig. 5 ).

### Scar Assessment

Scar evaluations using the VSS were conducted preoperatively and again at 12 months following lipografting. The results demonstrated scar improvement in all five patients, with pliability being the most consistently improved parameter. Compared with preoperative evaluations, all scars were more pliable at 12 months post-lipografting. Also, all scars were scored as flat after 12 months, likely reflecting tissue leveling through volume restoration of the surrounding tissue and improved scar quality from the regenerative effects of fat graft. The total VSS score for scars decreased from an initial mean of 3.6 points preoperatively to a maximum of 2 points at 12 months ( Table 3 ).

**Table 3:** This table shows the Vancouver Scar Scale scores for each patient, recorded preoperatively and after 12 months

	VSS preoperatively					VSS after 12 months				
Patient	Vascularity	Pigmentation	Pliability	Height	Total	Vascularity	Pigmentation	Pliability	Height	Total
1	0	2	2	0	**4**	0	0	1	0	**1**
2	0	2	2	1	**5**	0	2	0	0	**2**
3	0	1	2	0	**3**	0	0	0	0	**0**
4	0	1	1	1	**3**	1	1	0	0	**2**
5	0	0	2	1	**3**	0	0	1	0	**1**

Abbreviation: VSS, Vancouver Scar Scale.

It evaluates the severity of scars in terms of pigmentation, vascularity, pliability, and height, comparing the scar characteristics before and after the 1-year follow-up period.

Additionally, to assess patient satisfaction with the procedure, we employed a simple 4-point scoring system: Unsatisfied, neutral, satisfied, and very satisfied. Four out of five patients reported being very satisfied with the procedure, while the remaining patient reported being satisfied. Notably, one patient mentioned improved tolerance for prolonged sitting following the intervention.

## Discussion

This study aims to enhance secondary prevention strategies for pressure ulcers in patients with SCIs. Pressure ulcers pose a lifelong challenge for SCI patients, with a high incidence rate despite preventive measures. When late-stage pressure ulcers develop, surgical debridement and reconstruction become the only treatment options. However, these procedures often leave scars that make the tissue more fragile and prone to further ulcers, as the persistent pressure on the affected area remains. Recurrence rates are alarmingly high. For example, Tsai et al reported a 67.4% recurrence rate in SCI patients, 17 and Morel et al found that half of the patients experienced recurrence after flap surgery, with one-third occurring at the surgical site. 18 These statistics highlight the ongoing difficulty of managing pressure ulcers even after successful surgical interventions.

Additionally, each reconstructive surgery consumes viable tissue, reducing options for future operations. In cases of multiple recurrences, reconstruction may become severely limited or even unfeasible. This underscores the critical need for improved secondary prevention strategies to reduce the need for complex reoperations. We propose that lipografting can provide additional padding over bony prominences, potentially protecting these areas from recurrence or extending the interval between surgeries. In our study, no signs of recurrence were observed during the 12-month follow-up period.

Postoperative care also plays a significant role. Following flap surgery, patients typically require strict bed rest. Lefèvre et al reported a regimen of 42 days in low-air-loss beds to support healing. 19 While effective for wound recovery, this prolonged immobilization is highly restrictive for patients and increases the risk of new pressure ulcers developing on contralateral sites. In contrast, preventive measures are less limiting. After lipografting in our study, patients were discharged just 1 day postsurgery with instructions to avoid pressure on the grafted area for 14 days. This protocol resulted in less than 50% fat graft resorption.

The economic burden of pressure ulcer treatment is significant. For SCI patients, it is estimated that pressure ulcer treatment accounts for up to one-quarter of their total care costs. 20 Therefore, improving prevention strategies not only reduces physical and emotional suffering but also alleviates the financial strain on both health care systems and patients. Current prevention measures, such as frequent repositioning 21 and pressure-relieving devices like specialized mattresses and cushions, 22 remain standard. Despite advancements in materials and techniques, the recurrence rates of pressure ulcers remain persistently high. Introducing an additional preventive tool like lipografting could decrease incidence and recurrence rates, reducing the need for invasive surgeries and associated costs.

Studies on fat graft retention have reported varying results, with retention rates ranging from 25–50% to as high as 80–90%. 23 For example, Wang et al documented a 51.8% resorption rate 6 months post-breast augmentation using MRI, 24 while Khouri et al reported a 79.8% retention rate at 9 months when breast preexpansion with Brava was employed, also measured using MRI. 25 However, most studies focus on healthy tissue in cosmetic applications. Limited data exist on fat retention in severely scarred or chronically altered tissue, such as that seen in pressure ulcer cases. One of the primary causes of pressure ulcers in paraplegic patients is chronic tissue hypoxia resulting from prolonged pressure that leads to capillary obstruction. Since fat graft retention is closely linked to the vascularity of the recipient site, it is expected that fat resorption would be higher in pressure-prone areas with reduced blood flow. In our study, the fat graft resorption rate was on average 39.2% at 12 months postoperatively, indicating that retention rates in this context are comparable to those seen in cosmetic procedures. This naturally leads to the question of what the main factors behind fat graft resorption in the recipient tissue might be.

In our experience, uniformly placing the graft into the recipient site is challenging, as the stiffness of scarred tissue hinders smooth cannula penetration. Performing extensive rigotomy may create a large open space, preventing the fat graft from maintaining close contact with the surrounding tissue. However, fat grafting may contribute to scar improvement not only through its volumetric effect but also via its regenerative potential. Adipose tissue contains ADSCs, which promote angiogenesis, modulate inflammation, and stimulate extracellular matrix remodeling—mechanisms that can enhance dermal structure and scar quality. In our series, tissue pliability was one of the most positively affected parameters, suggesting that repeated fat grafting might gradually condition the fibrotic recipient bed, making it more receptive to subsequent grafting and potentially improving long-term outcomes.

Several methods have been used to assess fat retention, each with its strengths and limitations. MRI, as used by Jung et al 26 and others, provides accurate measurements but is costly and not always readily available. The water displacement method, while simple, lacks objectivity and is limited to protruding body parts like the breast. 27 Three-dimensional imaging (e.g., VECTRA) is another option, but it is best suited for specific body regions. 28 Ultrasonography, used in gluteal region studies, 29 offers an accessible and reliable alternative, provided it is performed by an experienced specialist, with consistency in examiners to minimize errors.

To evaluate the scar quality, the VSS was selected for its simplicity, reproducibility, and focus on key clinical features such as vascularity, pigmentation, pliability, and height. While the Patient and Observer Scar Assessment Scale (POSAS) offers the advantage of combining objective and subjective evaluations, and may be more sensitive to features such as scar height or surface irregularities, we opted for VSS given the small sample size and the need for a standardized, clinician-reported tool. The VSS certainly has its limitations, particularly in assessing scar height, as it lacks a specific rating for depressed scars. Future studies may benefit from incorporating complementary scales like POSAS to provide a more comprehensive assessment of scar outcomes.

While the small sample size of five patients limits the statistical power of this study and precludes broad generalizations, the findings remain clinically valuable. The limited cohort increases the risk of type II errors and reduces the ability to detect more subtle effects; however, as a pilot study, it provides important preliminary data and practical insights into the feasibility, safety, and potential benefits of fat grafting in pressure-prone areas. A larger sample would provide more robust results. Additionally, the follow-up period was 12 months. While our results showed no signs of pressure ulcer recurrence and improved tissue quality, the long-term behavior of the grafted tissue remains unknown. Questions remain about whether the tissue would thin further over time and when the procedure might need to be repeated. Given the chronic nature of the condition, a longer follow-up of 2 to 3 years would be necessary to assess the durability of results and determine whether additional fat grafting is needed. Determining the optimal timing for repeat procedures warrants further investigation.

### Conclusion

In conclusion, this study demonstrates that lipografting is a promising adjunct to secondary prevention of pressure ulcers in SCI patients. It provides additional tissue padding, reduces recurrence rates, and is less restrictive than traditional postoperative protocols. However, further research is needed to confirm its long-term efficacy and explore strategies for broader implementation.
